# Supplementary material for: A Comprehensive Evaluation of Associations Between Routinely Collected Staging Information and The Response to (Chemo)Radiotherapy in Rectal Cancer
Source: Cancers (Basel). 2020 Dec 22;13(1):16. doi: 10.3390/cancers13010016 (PMC7792936; doi:10.3390/cancers13010016)
Supplement: Supplementary file 1 [file cancers-13-00016-s001.pdf]

Supplementary Materials: A Comprehensive Evaluation of Associations Between Routinely Collected Staging Information and The Response to (Chemo)Radiotherapy in Rectal Cancer

Klara Hammarström, Israa Imam, Artur Mezheyeuski, Joakim Ekström, Tobias Sjöblom and Bengt Glimelius

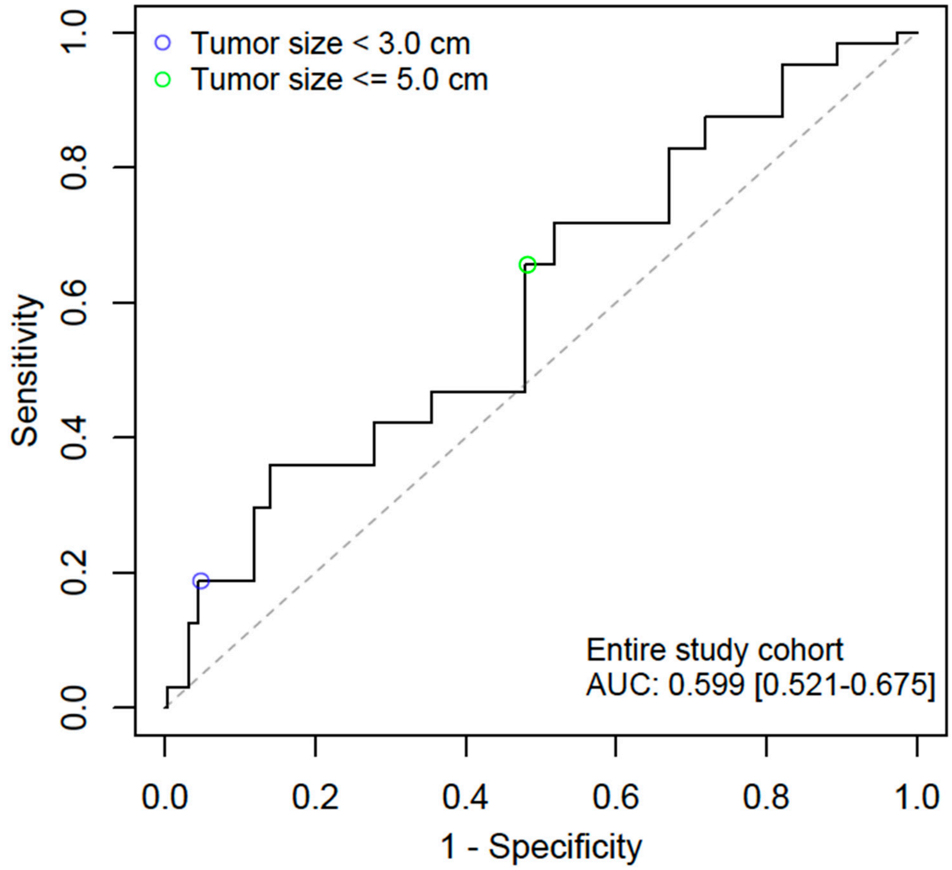

Figure S1. Receiver operating characteristic (ROC) curve of tumor size in the entire study cohort.

ROC curves of tumor size (solid line) and the reference uninformative random classifier (dashed line), both used toward predicting complete remission (CR). When applying a 3.0 cm cut-off for tumor size, observed sensitivity is 18.8% and specificity 95.2% (blue circle), and when applying a 5.0 cm cut-off for tumor size then observed sensitivity is 65.6% and specificity 51.8% (green circle). Annotated with area under curve (AUC) value and its 95% confidence interval.

Table S1. Clinical tumor stage, size (length) and CRP and their associations to treatment response in the entire cohort and scRT, CRT and scRT+CT groups.

	**Total Cohort** *n* = 383			**scRT + DELAY/W&W** *n*= 155			**CRT + DELAY/W&W** *n* = 94			**scRT+CT + DELAY/W&W** *n* = 134		
	**CR**	**NON-CR**	**P-VALUE** [A]	**CR**	**NON-CR**	**P-VALUE** [A]	**CR**	**NON-CR**	**P-VALUE** [A]	**CR**	**NON-CR**	**P-VALUE** [A]
TUMOR STAGE [B]			0.007			0.006			<0.001			0.096
cT1-2	12 (46)	14 (54)		5 (28)	13 (72)		6 (100)	0		1 (50)	1 (50)	
cT3a	4 (15)	22 (85)		2 (12)	15 (88)		0	3 (100)		2 (33)	4 (67)	
cT3b	13 (20)	53 (80)		4 (11)	33 (89)		2 (22)	7 (78)		7 (35)	13 (65)	
cT3c	12 (14)	75 (86)		0	30 (100)		3 (14)	18 (86)		9 (25)	27 (75)	
cT3d	2 (8)	22 (92)		0	6 (100)		2 (18)	9 (82)		0	7 (100)	
cT4a	3 (7)	38 (93)		0	10 (100)		1 (11)	8 (89)		2 (9)	20 (91)	
cT4b	18 (17)	89 (83)		0	31 (100)		3 (9)	32 (91)		15 (37)	26 (63)	
MISSING [C]	1	5		1	5		0	0		0	0	
TUMOR SIZE												
5 cm			0.011			0.534			0.180			0.043
≤5 cm	42 (22)	151 (78)		7 (9)	70 (91)		11 (23)	36 (77)		24 (35)	45 (65)	
>5 cm	22 (12)	162 (88)		4 (5)	70 (95)		6 (13)	41 (87)		12 (19)	51 (81)	
MISSING [C]	1	5		1	3		0	0		0	2	
4			0.026			0.177			0.035			0.163
≤4 cm	27 (24)	88 (76)		6 (11)	48 (89)		8 (32)	17 (68)		13 (36)	23 (64)	
>4 cm	37 (14)	225 (86)		5 (5)	92 (95)		9 (13)	60 (87)		23 (24)	73 (76)	
MISSING [C]	1	5		1	3		0	0		0	2	
3 cm			<0.001			<0.001			0.005			0.047
≤3 cm	12 (44)	15 (56)		5 (28)	13 (72)		3 (100)	0		4 (67)	2 (33)	
>3 cm	52 (15)	298 (85)		6 (5)	127 (95)		14 (15)	77 (85)		32 (25)	94 (75)	
MISSING [C]	1	5		1	3		0	0		0	2	
CRP			0.088			1.000			1.000			0.031
≤ 10 MG/L	45 (19)	193 (81)		7 (8)	86 (92)		14 (20)	57 (80)		24 (32)	50 (68)	
> 10 MG/L	7 (10)	62 (90)		3 (8)	33 (92)		2 (22)	7 (78)		2 (8)	22 (92)	
MISSING	13	63		2	24		1	13		10	26	

Results are given as numbers, *n* (%). Complete responders (CR) and non-complete responders (non-CR) were compared in the statistical analyses [A] Chi-square test of independence, Fisher's exact test was used if more than 20% of observations have expected frequencies < 5. [B] Based upon MRI in 372 patients and upon other information (mainly computed tomography (CT) and/or palpation) in 11 patients [C] Patients with no information on the variable in question have been excluded in the statistical analyses. Abbreviations: CRP = C-reactive protein; scRT = short-course radiotherapy; CRT = chemotherapy; CT = chemotherapy; W&W = watch-and-wait.

Copyright: © 2020 by the author. Licensee MDPI, Basel, Switzerland. This article is an open access article distributed under the terms and conditions of the Creative Commons Attribution (CC BY) license (http://creativecommons.org/licenses/by/4.0/).
